# A genome-wide association study in Swedish colorectal cancer patients with gastric- and prostate cancer in relatives

**DOI:** 10.1186/s13053-024-00299-z

**Published:** 2024-11-14

**Authors:** Johanna Samola Winnberg, Litika Vermani, Wen Liu, Veronika Soller, Jessada Thutkawkorapin, Mats Lindblad, Annika Lindblom

**Affiliations:** 1 Division of Surgery, Department of Clinical Science Intervention and Technology (CLINTEC), Karolinska Institutet, Stockholm, Sweden; 2 Department of Upper Abdominal Diseases, Karolinska University Hospital, Stockholm, Sweden; 3 Karolinska University Hospital Huddinge, Stockholm, 141 86 Sweden; 4 Department of Molecular Medicine and Surgery, Karolinska Institutet, Stockholm, Sweden; 5 Department of Surgical Sciences, Functional Pharmacology and Neuroscience, Uppsala University, Uppsala, Sweden; 6 Department of Computer Engineering, Faculty of Engineering, Chulalongkorn University, Bangkok, 10330 Thailand; 7 Department of Clinical Genetics, Karolinska University Hospital, Stockholm, Sweden; 8 K1 MMK Clinical Genetics, Stockholm, 171 76 Sweden

**Keywords:** GWAS, Hereditary cancer, Colorectal cancer, Gastric cancer, Prostate cancer, Cancer syndrome, Inherited, Familial, Genetic, NGS

## Abstract

**Background:**

A complex inheritance has been suggested in families with colorectal-, gastric- and prostate cancer. Therefore, we conducted a genome-wide association study (GWAS) in colorectal cancer patients, who’s relatives had prostate-, and/or gastric cancer.

**Methods:**

The GWAS analysis consisted of 685 cases of colorectal cancer and 4780 healthy controls from Sweden. A sliding window haplotype analysis was conducted using a logistic regression model. Thereafter, we performed sequencing to find candidate variants, finally to be tested in a nested case–control study.

**Results:**

Candidate loci/genes on ten chromosomal regions were suggested with odds ratios between 1.71–3.62 and *p*-values < 5 × 10–8 in the analysis. The regions suggested were 1q32.2, 3q29, 4q35.1, 4p15.31, 4q26, 8p23.1, 13q33.3, 13q13.3, 16q23.3 and 22q11.21. All regions, except one on 1q32.2, had protein coding genes, many already shown to be involved in cancer, such as *ZDHHC19, SYNPO2, PCYT1A, MYO16, TXNRD2, COMT,* and *CDH13*. Sequencing of DNA from 122 colorectal cancer patients with gastric- and/or prostate cancer in their families was performed to search for candidate variants in the haplotype regions. The identified candidate variants were tested in a nested case–control study of similar colorectal cancer cases and controls. There was some support for an increased risk of colorectal-, gastric-, and/or prostate cancer in all the six loci tested.

**Conclusions:**

This study demonstrated a proof of principle strategy to identify risk variants found by GWAS, and identified ten candidate loci that could be associated with colorectal, gastric- and prostate cancer.

**Graphical Abstract:**

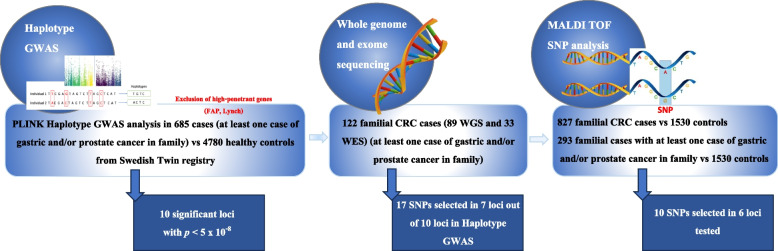

**Supplementary Information:**

The online version contains supplementary material available at 10.1186/s13053-024-00299-z.

## Introduction

Cancer has multi-factorial aetiology, that is not yet fully understood. Lifestyle factors, environment, as well as genetics play a role. Today, several germline disease-causing variants in high-penetration genes are recognized. These are disease-causing variants in one of the DNA mismatch repair genes (Lynch’s Syndrome), *BRCA1* or *BRCA2* (Hereditary Breast and Ovarian Cancer Syndrome), *CDH1* (Hereditary Diffuse Gastric Cancer), *TP53* (Li-Fraumeni Syndrome), *APC* (Familial Adenomatous Polyposis) and *STK11* (Peutz-Jeghers Syndrome), to mention a few associated with colorectal cancer, gastric cancer and/or prostate cancer [1–3]. Despite this knowledge, it has been difficult to explain, why some cancers run in families that do not carry any of the known disease-causing variants. Instead, one has started to consider cancer as a disease with complex traits [4]. There has been a shift in gene discovery efforts from models of predisposition based on high-penetrance single-gene variants to polygenic models, studied in genome-wide association studies (GWAS) [5].

Many GWAS have been carried out to find loci for different cancer types [2, 6–10] These still cannot explain the majority of familial cancer and therefore, one has started to look for loci that predispose to more than one type of cancer [11].

We have previously searched for new cancer syndromes and have suggested a syndrome involving families with colorectal- and other cancers, and most important, gastric- and prostate cancer [12]. Linkage analysis in families with colorectal-, gastric- and prostate cancer was carried out. No high penetrant disease-causing variant was found, and instead a complex disease was suggested for this syndrome [13]. Thus, a GWAS on colorectal cancer (CRC) cases from families with both colorectal-, prostate-, and/or gastric cancer was designed. In this study, controls consisted of healthy individuals from all Sweden, recruited from the Swedish twin registry [14]. Thereafter, next generation sequencing (NGS) in patients aimed to find candidate variants in the suggested loci. Finally, a case–control study in patients and healthy controls was undertaken to find support for these ten variants in the haplotype regions.

## Materials and methods

### Cases and controls for GWAS

Colorectal cancer cases were selected from a multi-centre study, the Colorectal Cancer Low-risk study [12]. The study recruited more than 3300 newly diagnosed colorectal cancer patients from 14 hospitals in the middle of Sweden, between 2004 and 2009. All patients provided written informed consent, and the study was approved by the regional research ethics committees in Stockholm 2002 (Stockholms Regionala Etikprövningsnämnd) and Uppsala (Uppsalas Regionala Etikprövningsnämnd), Dnr: 02–489 and 03–114.

Cancer occurrences in first- and second-degree relatives, as well as cousins were recorded. FAP cases and Lynch’s syndrome were excluded based on pathology report, family history and microsatellite instability (MSI) testing to avoid families with germline disease-causing variants in high-penetrant genes. Patients with at least one more close relative with colorectal cancer, were coded as familial colorectal cancer. Patients with at least one case of gastric- or prostate cancer within their family, were selected for the studies. In total, 685 of 2663 genotyped patients were included as cases (Table 1).

**Table 1:** Family history of patients/families in the three experiments: GWAS, direct sequencing, and Maldi-tof + association study

	**1. GWAS; 685 cases (510 sporadic)**	**2. SEQ; 122 cases (all familial)**	**3. Association; 827 cases (all familial)**	**Sub cohort; 293 cases (all familial)**
**Number of cancer cases among relatives to the index patients in the different experiments**				
**Breast**	276	52	262	110
**Pancreatic**	36	8	49	18
**Gastric**	523	123	216	211
**Prostate**	468	96	201	193
**Gynaecologic**	143	27	161	58
**Biliary tract**	21	3	15	9
**Lung**	106	14	112	44
**Bladder**	28	3	38	13
**Leukaemia/lymphoma**	91	25	120	49
**Skin/melanoma**	61	19	58	34
**CNS**	59	11	72	26
**Renal**	35	4	36	14
**Other**	395	70	459	179

Controls for GWAS were selected from the Swedish Twin Registry [14] and consisted of 4780 healthy individuals. Phenotypic data on cancer had previously been obtained through linking the twins to the Swedish Cancer Registry using the unique person identification number available for all Swedish citizens. Only one twin from each twin pair was included in the analysis. In cases where one of the twins had cancer, both twins were excluded from the study. No information on family history was available for controls.

### Genotyping and quality control for GWAS

DNA was extracted from peripheral blood samples for both cases and controls. The cases from the Colorectal Cancer Low-risk study were genotyped at the Center for Inherited Disease Research at Johns Hopkins University, US using the Illumina Infinium® OncoArray-500 K [15]. Controls from the Swedish TwinGene registry were genotyped in Uppsala, Sweden using the Illumina OmniExpress bead chip or the Illumina Infinium PsychArray-24 BeadChip [16]. All samples underwent a quality control (QC1) at each genotyping centre. The data was merged, 240,370 SNPs (single nucleotide polymorphism) were shared between the two platforms and TOP strand format was accounted for. In the analysis we used only genotyped SNPs. Imputed SNPs were not used since imputation might miss typical Swedish haplotypes.

In the second QC round (QC2), heterozygous haploid genotypes were excluded as well as samples with gender inconsistency and same position variants which meant that 239,113 SNPs and 7472 individuals (2690 cases and 4782 controls) passed QC2 [17]. A third QC stage (QC3) was performed on the merged data, where SNPs with < 98% call rate, < 1% minor allele frequency (MAF) and those inconsistent with Hardy–Weinberg (hwe 0.001) equilibrium in controls were removed, and 224,210 SNPs remained after QC3. In the fourth and final QC (QC4) a multidimensional scaling (MDS) analysis was conducted on all the remaining markers for the purpose of population stratification and to identify ethnic outliers. These outliers were excluded from the dataset. After QC4, 219,114 SNPs and 7417 individuals (2637 cases, 4780 controls) remained to perform further downstream analyses. Finally, for the GWAS we selected from the 2637 CRC cases, 685 with gastric- and/or prostate cancer in their families, and all 4780 controls.

### Haplotype GWAS

A logistic regression model was employed to examine the association between one single SNP, or a haplotype, and cancer risk. Corresponding Odds Ratios (OR), standard errors, 95% confidence intervals (CI) and P-values were calculated accordingly using PLINK v1.07 [18]. When running plink, the following parameters were requested: “hap-logistic” (haplotype logistic regression analysis), “hap-window 1–25” (sliding window sizes 1 to 25) and default settings. That includes haplotypes phasing with the E-M algorithm, minor haplotype frequency of 0.01 and omnibus association test. As p-value criteria for genome-wide statistical significance, p < 5 × 10^−8^ was used. Haplotypes describe the linear relationship of a series of loci along the chromosome strand, and in PLINK defined by a certain number of single SNP markers. A sliding-window haplotype analysis tested more than 8 million sliding windows, which means that each SNP is involved numerous times involving 24 SNPs upstream and 24 SNPs downstream. No adjustments were made for gender or age. To determine what windows to use for haplotype analysis, we previously tested different window sizes and found that windows with more than 25 SNPs rarely showed positive results [16]. Thus, windows 1 and 2–25 were chosen for analysis. Quantile–quantile (QQ) plot (supplemental Fig. 1 and [17]) was performed and observed p-values in all samples were compared to those expected for a null distribution. The QQ plot was generated in R using the qqman package.

### Cases for sequencing to find candidate SNPs in each GWAS locus

From the 685 patients used in GWAS, 89 familial CRC cases with the most gastric- and/or prostate cancer in their families, were selected for whole genome sequencing (WGS). Another set of 33 familial CRC cases with gastric- and prostate cancer in their families, already used in a previous study [19], could also be included for the next experiment, in order to search candidate SNPs to test using association analysis (Table 1).

### Whole genome sequencing analysis

Genomic DNA was extracted using Gentra Puregene Blood Kit (Qiagen) according to the manufacturer’s protocol, followed by the quantification using Qubit Fluorometer (Life Technologies). The sequencing libraries were prepared using Illumina TruSeq PCR-free kit (Illumina) with average coverage of 30X. The sequencing libraries were prepared according to the manufacturer’s protocol (Illumina). In short, genomic DNA from 89 samples was fragmented using Covaris and subjected for library preparation involving end-repair, followed by A-tailing and adaptor ligation. The sequencing was performed on NovaSeq6000, and data analyzed with the Sarek germline pipeline [20].

### Cases and controls for association study

For the final case–control study of candidate variants, 827 familial colorectal cancer cases could be used. Those represented 691 familial CRC cases from the low-risk study, and 136 familial cases recruited from the department of Clinical Genetics. Among them were 293 CRC cases, with gastric- and prostate cancer in their families. As 1530 healthy controls were used: 540 healthy spouses from the low-risk study and thus the same region as the 827 (Stockholm-Uppsala in the middle of Sweden), as well as 990 blood donors from the Stockholm region.

### Algorithm for selection of candidate SNPs using the sequencing data

The GWAS data was in hg37 reference genome (GRCh37) and the sequencing data in hg38 reference genome (GRCh38). The base pair positions for each of the loci were converted to hg38 (Ensembl genome browser 110) to be able to search for candidate variants in the sequencing data. To find genes, regulatory elements, non-coding regions or pseudogenes, the haplotype region was searched in Ensembl genome browser 110. It was followed by a search for the candidate variants in the genes or other regions in the sequencing data (WGS and WES). Filtering was done as follows: each chromosome was sorted based on the positions (smallest to largest) and studying for the gene variant in each haplotype region. Synonymous variants and variants without known allele frequency were not considered. To be able to test with MALDI-TOF (Matrix Assisted Laser Desorption-Ionization-Time of Flight) using MassARRAY Platform, we selected only SNPs. Allele frequencies were taken from gnomAD (version v.3.1.2), SweGen Variant Frequency database (SweFreq) and the frequencies in the 122 sequenced samples were also calculated. Rare variants (< 0.005), and common variants (> 15%) were filtered out.

### Association study using MALDI-TOF

SNP genotyping was performed on MassARRAY Platform from Agena based on MALDI-TOF analysis. The genotyping was done in the core facility at Translational Analysis in Molecular Medicine (TAMM) at the Karolinska University Hospital. The steps involved primers design using software package from Agena, PCR amplification of the desired SNP loci, clean up using SAP enzyme, extension reaction and fragment analysis on Agena MassARRAY analyzer. Agena’s SpectroTyper software was used for automated allele calling, followed by validation using human DNAs from the CEU population genotyped by the Hapmap consortium (CEU panel). In all steps, positive and negative controls were used. Some of the samples were repeated to ensure reproducibility of the assay. Association testing was performed for each individual SNP separately. OR was manually calculated using the genotype count in cases and controls; OR > 1 was considered associated with the increased disease risk.

## Results

A haplotype GWAS using windows 1–25 was undertaken, with 685 CRC cases and a large set of controls from the Swedish Twin Registry. A Manhattan plot from the single SNP analysis is shown in the supplemental Fig. 2. To ensure that every region in the genome was included in the analysis, the sliding window strategy was used. All possible haplotypes (size 1–25 SNPs) were generated, and we chose for our study only haplotypes with a positive OR for risk and searched for haplotypes with p < 5 × 10^–8^. Ten haplotypes in ten different loci reached a level of p < 5 × 10^–8^. The loci were 1q32.2, 3q29, 4q35.1, 4q26, 4p15.31, 8p23.1, 13q33.3, 13q13.3, 16q23.3, 22q11.21 (Table 2, supplemental Tables 1–23). ORs were between 1.71 and 3.62 (Table 2).

**Table 2:** Results GWAS

Locus	HS	BP 1	BP2	HF	OR	P Value	Genes
1q32.2	9	208,968,409	209,083,609	0.03	2.08	3.17E-08	No gene
3q29	17	195,750,742	195,973,244	0.02	2.99	2.32E-08	*TFRC*, *SLC51A*, *ZDHHC19*, *PCYT1A*
4q35.1	21	185,088,648	185,252,818	0.01	2.84	1.30E-08	*ENPP6*
4q26	20	119,506,139	119,835,148	0.01	3.62	1.59E-08	*METTL14*, *SEC24D*, *SYNPO2*
4p15.31	24	20,852,244	21,112,046	0.01	3.25	2.72E-08	*KCNIP4*
8p23.1	14	11,236,975	11,355,821	0.01	3.04	4.47E-08	*FAM167A*, *BLK*
13q33.3	13	109,832,287	109,897,922	0.11	1.71	9.20E-09	*MYO16*
13q13.3	9	37,374,156	37,460,648	0.07	1.86	4.15R-08	*RFXAP*, *SMAD9*
16q23.3	8	82,871,769	82,899,877	0.01	3.60	1.38E-08	*CDH13*
22q11.21	12	19,872,009	19,930,121	0.03	2.30	2.56E-10	*TXNRD2*, *COMT*

*HS* Haplotype window sizes, *BP* Base pair (GRCh37), *HF* Haplotype frequency in samples, *OR* Odds ratio

Next, 89 samples from CRC patients underwent WGS to search for candidate risk variants in the defined regions of the ten loci. Sequencing results from those 89 patients, as well as 33 patients from a previous study of WES in familial CRC, were used for the search. Altogether, 17 variants from 7 loci (rs754397679, rs41298105, rs41299376, rs181290971, rs141180741, rs527897389, rs35392900, rs184578242, rs191831989, rs73872825, rs35657205, rs17054519, rs118015060, rs41275074, rs56393169, rs117287159 and rs72807847) fulfilling the criteria were tested to find markers to be used for the final association analysis. All familial CRC patients and controls were sent for genotyping of these 17 markers using MALDI-TOF analysis. In the end, only 10 out of the 17 variants from 6 loci (3q29, 4q26, 4q35.1, 13q13.1, 13q33.3 and 16q23.3) remained after the procedure of MALDI-TOF and could be analysed in the association study [21].

First, the association study used 827 familial CRC cases and 1530 controls. Although all six loci had markers with OR > 1, there were no statistically significant results (Table 3). To further evaluate the hypothetical syndrome, a second association study was performed in a sub-cohort of 293 familial CRC samples from families with colorectal-, gastric- and prostate cancer, and the same controls (Table 3).

**Table 3:** Results from association studies in all familial CRC (cases A) and sub-cohort of families with colorectal-, gastric- and prostate cancer (cases B)

Locus	SNP	Gene	Type	Ref	Alt	Cases A	Controls	OR	*p*	Cases B	OR	*p*
**3q29**	**rs181290971**	***PCYT1A***	**3' UTR**	G	A	808	1510	0.83	0.66	283	**1.06**	0.90
**3q29**	**rs41299376**	***TFRC***	**3' UTR**	T	A	819	1526	1.25	0.26	293	**1.61**	0.07
3q29	rs754397679	*PCYT1A*	missense	C	T	785	1447	0	0	284	0	0
4q26	rs141180741	*SEC24D*	missense	G	A	818	1514	0.83	0.66	293	1.08	0.82
4q26	rs184578242	*METTL14*	3' UTR	A	G	819	1527	0.51	0.12	293	0.62	0.43
**4q26**	**rs35392900**	***SEC24D***	**missense**	G	C	813	1505	0.87	0.64	292	**1.14**	0.72
4q35.1	rs73872825	*ENPP6*	intron	A	G	811	1473	1.14	0.50	292	1.06	0.85
**13q13**	**rs118015060**	***SMAD9***	**3' UTR**	A	G	819	1527	1.02	0.92	293	**1.39**	0.27
**13q33.3**	**rs56393169**	***MYO16***	**3' UTR**	T	C	819	1526	1.08	0.51	293	**1.24**	0.19
**16q23.3**	**rs72807847**	***CDH13***	**missense**	A	G	819	1527	1.40	0.37	293	**1.63**	0.33

Markers supporting the hypothesis in bold

*SNP* single nucleotide polymorphism, *OR* odds ratio, *p* = *p*-value

The number of samples was small, and no results were statistically significant. However, OR was above one for all six loci; five of six had a higher ORs in the sub-cohort analysis. The results supported an increased risk of cancer caused by the candidate variants in the selected families.

## Discussion

A haplotype GWAS focusing on CRC associated with gastric- or prostate cancer, identified altogether 10 candidate loci with the selected p-value criteria p < 5 × 10^–8^. ORs were higher than usually seen in GWAS [6–10], and different from and higher than the previous haplotype GWAS on all unselected CRC cases and controls [17]. This could be interpreted to support the hypothesis of risk markers associated with CRC and other tumours such as gastric- and prostate cancer. Another explanation for higher OR in haplotype studies could be that the haplotypes often involve more than one gene and thus more than one disease causing variant could act to increase the risk at this locus. Most published SNPs from GWAS suggested few genes in contrast to haplotype GWAS, where genes are found in most of the suggested haplotypes. The fact that few genes were suggested in most SNP GWAS could be because the assumed target risk variant at a specific locus could be quite far from the suggested SNP, while a haplotype spans over a certain distance and is more likely to include one or more candidate genes.

In an attempt to find the putative risk associated variants in the regions, sequencing of 122 CRC cases with gastric- and/or prostate cancer in their families was done to find candidate variants in these loci. It was only possible to finally test 6 of the loci (17 markers from 7 loci were first identified and 10 could be tested), and there was some support for all six loci, although the limited number of available cases and controls might explain the lack of statistically significant results. ORs were lower than those from the GWAS. This could be explained if the markers tested were not the actual functional risk SNPs. It was not affordable to test all samples used in the GWAS and only 89 of the 685 cases in the GWAS were sequenced, and the candidate haplotype frequencies were low, why it was unlikely to identify all putative risk variants suggested from the GWAS in the 122 cases sequenced. Moreover, the GWAS and the final association study did not use the exact same samples. In the GWAS, both familial and sporadic CRC cases were used, and all fulfilled the criteria of having at least one gastric- or prostate cancer case among close relatives, while in the final association analysis, mostly familial CRC cases were used.

Almost all genes suggested here have been implicated in cancer. Several of the candidate genes above are related to known cancer signalling pathways. The wnt/betacatenin pathway, here represented by the gene (*ZDHHC19*), is well known to be involved in CRC [22]. However, this pathway has also been implemented in both prostate- and gastric cancer [23, 24]. Two genes (*SYNPO2, PCYT1A*) act in the Pi3K/Akt/mTOR pathway, involved in carcinogenesis of many tumours including colorectal-, gastric- and prostate cancer [25–27]. *MYO16* has been suggested as one candidate after linkage analysis in familial breast cancer [28] and *MYO16-AS* in the same haplotype has been described to act in both bladder and lung cancer [29, 30]. *TXNRD2,* thioredoxin reductase 2, a known selenoprotein and DNA damage response gene, is implicated in cancer, such as prostate cancer [31] and colorectal cancer [32]. The *COMT*, coding for the enzyme catechol-O-methyltransferase, functions to degrade catecholamines, catecholoestrogens and various drugs and substances with a similar structure. *COMT* plays a role in both colorectal- [33], gastric- [34] and prostate cancer [35], but has also been published in relation to many other neoplasms. Some other candidate genes are less well studied but associated to CRC and gastric- as well as prostate cancer: *TFRC* [36–38]. Three of the candidate genes code for TMEM proteins, suggested to be implicated in cancer [39]. *SMAD9* and *ENPP6* are both involved in bone mineralization and could be involved in cancer like the gene *BMPR1A* (bone mineralization protein 1A), where variants predispose to CRC [1]. *CDH13* (also known as T-cadherin) at locus 16q23.3 is involved in several neoplasms, besides CRC, prostate- and gastric cancer [40]. *CDH13* is interesting also in the context that another gene in the same family, *CDH1*, is responsible for familial early onset diffuse gastric cancer [2].

The fact that many of the genes have been implicated also in other cancers, besides those selected for the study, further supports an increased cancer risk of varying degree for different tumours. This is similar in many cancer syndromes, e.g., Lynch’s syndrome, where there is an increased risk of colorectal-, but also other tumours. One limitation of the study is that only CRC cases were analysed, and it would be of interest to study also gastric- and prostate cancer families with CRC in close relatives. However, the design in GWAS of CRC cases with gastric- or prostate cancer in their families, and the two-step procedure in the final association analysis still suggested markers with an increased risk for all three tumour types.

## Conclusions

In conclusion, we consider the study as a proof of principle; it is possible to use the design in this paper to find SNPs associated with disease in risk haplotype regions. Moreover, our study identified candidate loci, -genes and -SNPs that could be associated with a modest increased risk of CRC, gastric- and prostate cancer. Further studies of these loci/genes are warranted to search for the causative variants, and to determine the actual risk at the loci.

## Supplementary Information

Supplementary Material 1: Figure S1: Quantile-quantile plot (QQ-plot). QQ-plot of observed and expected *P*-values for single SNP analysis, -log 10 transformed. The diagonal red line represents the expected null hypothesis (= no association). Figure S2: Manhattan plot. Observed *P* values along the chromosomes for SNP association. The blue line represents suggestive statistical significance, *p*<5x10^−5^. Table S1-S23.

## Data Availability

No datasets were generated or analysed during the current study.

## Abbreviations

CRC: Colorectal cancer
CI: Confidence interval
FAP: Familial adenomatous polyposis
GC: Gastric cancer
GWAS: Genome-wide association study
HF: Haplotype frequency
HS: Haplotype window size
MSI: Microsatellite instability
MAF: Minor allele frequency
MALDI-TOF: Matrix Assisted Laser Desorption-Ionization-Time of Flight
MDS: Multidimensional scaling
NGS: Next generation sequencing
OR: Odds ratio
PrC: Prostate cancer
QC: Quality control
QQ plot: Quantile–quantile plot
SNP: Single nucleotide polymorphism
WES: Whole exome sequencing
WGS: Whole genome sequencing
