# Supplementary figures and images for: A genome-wide association study in Swedish colorectal cancer patients with gastric- and prostate cancer in relatives
Source: Hered Cancer Clin Pract. 2024 Nov 14;22:25. doi: 10.1186/s13053-024-00299-z (PMC11562479; doi:10.1186/s13053-024-00299-z)

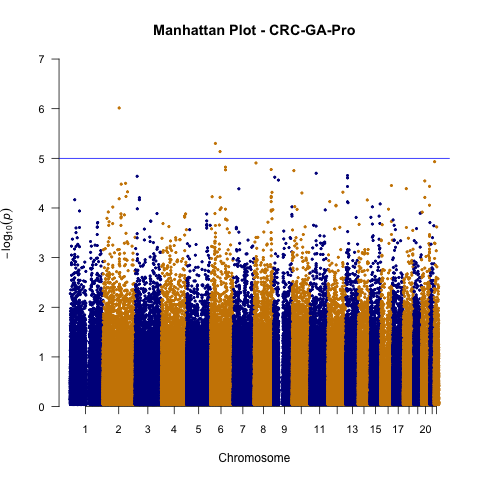

Supplement: Supplementary file 1 — Supplementary Material 1: Figure S1: Quantile-quantile plot (QQ-plot). QQ-plot of observed and expected P-values for single SNP analysis, -log 10 transformed. The diagonal red line represents the expected null hypothesis (= no association). Figure S2: Manhattan plot. Observed P values along the chromosomes for SNP association. The blue line represents suggestive statistical significance, p<5x10^−5^. Table S1-S23. [file 13053_2024_299_MOESM1_ESM.zip › Manhattanplot_singles.png]

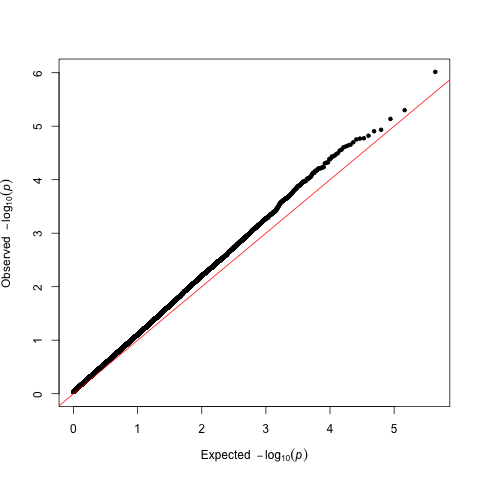

Supplement: Supplementary file 1 — Supplementary Material 1: Figure S1: Quantile-quantile plot (QQ-plot). QQ-plot of observed and expected P-values for single SNP analysis, -log 10 transformed. The diagonal red line represents the expected null hypothesis (= no association). Figure S2: Manhattan plot. Observed P values along the chromosomes for SNP association. The blue line represents suggestive statistical significance, p<5x10^−5^. Table S1-S23. [file 13053_2024_299_MOESM1_ESM.zip › QQplot_singles.png]
